# Skin‐resident memory T cells as a potential new therapeutic target in vitiligo and melanoma

**DOI:** 10.1111/pcmr.12803

**Published:** 2019-07-08

**Authors:** Marcella Willemsen, Rugile Linkutė, Rosalie M. Luiten, Tiago R. Matos

**Affiliations:** ^1^ Department of Dermatology and Netherlands Institute for Pigment Disorders Amsterdam University Medical Centers, University of Amsterdam, Cancer Center Amsterdam Amsterdam Infection & Immunity Institute Amsterdam the Netherlands

**Keywords:** autoimmunity, immunology, immunotherapy, melanoma, T lymphocytes, vitiligo

## Abstract

Tissue‐resident memory T (T_RM_) cells are abundant in the memory T cell pool and remain resident in peripheral tissues, such as the skin, where they act as alarm sensors or cytotoxic killers. T_RM_ cells persist long after the pathogen is eliminated and can respond rapidly upon reinfection with the same antigen. When aberrantly activated, skin‐located T_RM_ cells have a profound role in various skin disorders, including vitiligo and melanoma. Autoreactive T_RM_ cells are present in human lesional vitiligo skin and mouse models of vitiligo, which suggests that targeting these cells could be effective as a durable treatment strategy for vitiligo. Furthermore, emerging evidence indicates that induction of melanoma‐reactive T_RM_ cells is needed to achieve effective protection against tumor growth. This review highlights seminal reports about skin‐resident T cells, focusing mainly on their role in the context of vitiligo and melanoma, as well as their potential as therapeutic targets in both diseases.

## 1 INTRODUCTION

The presence of a pathogen stimulates naïve T cells to differentiate into memory and effector T cells in order to eliminate pathogen‐infected cells. Memory T cells can be subgrouped into central memory T (T_CM_) cells, effector memory T (T_EM_) cells, and migratory memory T (T_MM_) cells (Sallusto, Lenig, Forster, Lipp, & Lanzavecchia, 1999; Watanabe et al., 2015). The T_CM_ cell pool predominates in secondary lymphoid organs and expresses markers, such as chemokine receptor CCR7 and the vascular addressin/L‐selectin (CD62L). In contrast, T_EM_ cells migrate into the non‐lymphoid tissues to clear the infection with their high cytokine production capacity and perforin expression. T_EM_ cells express low CCR7 and CD62L levels, but can express high levels of the tissue‐homing addressin and E‐selectin ligand cutaneous lymphocyte antigen (CLA), which enables them to enter into the skin (Farber, Yudanin, & Restifo, 2014; Mueller, Gebhardt, Carbone, & Heath, 2013). Expression of CCR7 and absence of CD62L characterize migratory memory T (T_MM_) cells, which recirculate between blood and tissues and are excluded from the lymph nodes (Watanabe et al., 2015).

Memory T cells were initially considered to be circulatory and to enter the tissues only when needed, to clear an infection. Work over the past years has defined another pool of memory T cells, called resident memory T (T_RM_) cells (Gebhardt et al., 2009; Masopust et al., 2010; Wakim, Waithman, van Rooijen, Heath, & Carbone, 2008). T_RM_ cells do not recirculate, but reside permanently in tissues such as skin, intestine, lung, brain, and female reproductive tract, where they provide rapid protective immunity against reinfecting pathogens (Jiang et al., 2012; Lefrancois & Masopust, 2002; Mueller & Mackay, 2016). Upon viral or bacterial infection, antigen‐specific primary and memory CD8^+^ T cells become present throughout the body. Resident memory CD8^+^ T cells isolated from non‐lymphoid tissues showed higher antigen‐specific response than circulatory memory cells isolated from lymphoid tissues (Masopust, Vezys, Marzo, & Lefrancois, 2001). T_RM_ can even respond more rapidly to tissue infection than circulatory memory cells (Ariotti et al., 2014; Clark, 2010; Schenkel et al., 2014). The T_RM_ cell population within each tissue is capable of recognizing the specific pathogens that most commonly affect those tissues, and T_RM_ cells remain in place long after pathogen elimination (Gebhardt et al., 2009; Jiang et al., 2012; Mackay et al., 2012).

Besides eliminating pathogens, T_RM_ cells may also contribute to various disorders when aberrantly activated. These cells can develop not only after pathogen infection, but also after sensitization to otherwise harmless environmental or self‐antigens (Clark, 2015). The involvement of T_RM_ cells has been demonstrated in various skin diseases, such as psoriasis (Cheuk et al., 2014; Clark, 2011; Matos et al., 2017; Suarez‐Farinas, Fuentes‐Duculan, Lowes, & Krueger, 2011), fixed drug eruptions (Shiohara, 2009), allergic contact dermatitis (Gaide et al., 2015; Honda, Egawa, Grabbe, & Kabashima, 2013), cutaneous T‐cell lymphoma—a malignancy of T_RM_ cells (Campbell, Clark, Watanabe, & Kupper, 2010) and vitiligo (Boniface et al., 2018; Cheuk et al., 2017; Richmond, Strassner, Rashighi, et al., 2018; Richmond, Strassner, Zapata, et al., 2018).

Interestingly, autoimmunity and tumor immunity are often linked, as exemplified by the association between vitiligo and melanoma. Overwijk et al. (2003) showed that the same specific lymphocytic response could promote tumor destruction and vitiligo, in the exact same mouse. Adaptive transfer of gp100‐specific CD8^+^ T cells in mice bearing B16 melanoma cured the mice of the tumor, but also caused vitiligo. The vitiligo started at the former tumor site, and even one year after therapy, these mice remained tumor‐free with progressive vitiligo. Gp100 is a member of a family of “self” (i.e., unmutated), melanoma/melanocyte differentiation antigens that are widely expressed by melanoma cells. Hence, vitiligo was caused by activated anti‐melanoma immunity that not only targeted malignant cells, but also healthy melanocytes. A subsequent study reported that tumor‐bearing mice with vitiligo generated 10‐fold larger CD8^+^ memory T‐cell populations that are specific for shared melanoma/melanocyte antigens than mice without vitiligo (Byrne et al., 2011). These responses were not observed in melanocyte‐deficient mice. CD8^+^ T cells in mice with vitiligo acquired phenotypic and functional characteristics of T_EM_ cells, suggesting that they were supported by ongoing antigen stimulation. Conversely, melanocyte‐deficient mice did not generate such protective responses, indicating a requirement for melanocyte destruction as antigen source in maintaining CD8^+^ T‐cell immunity to melanoma.

In humans, it has been observed that vitiligo can occur in melanoma patients spontaneously or during immunotherapy treatment and correlates with prolonged survival (Boasberg et al., 2006; Gogas et al., 2006; Quaglino et al., 2010; Teulings et al., 2015). Conversely, vitiligo patients have threefold lower probability of developing melanoma during their life span than non‐vitiligo patients (Paradisi et al., 2014; Teulings et al., 2013). Recent work has indicated the pathogenic involvement of T_RM_ cells in human vitiligo (Boniface et al., 2018; Cheuk et al., 2017; Richmond, Strassner, Rashighi, et al., 2018; Richmond, Strassner, Zapata, et al., 2018) and data on this are still emerging. Other studies have demonstrated a protective role for T_RM_ cells in melanoma (Boddupalli et al., 2016; Edwards et al., 2018; Enamorado et al., 2017; Gálvez‐Cancino et al., 2018; Malik et al., 2017; Murray et al., 2016; Park, Buzzai, et al., 2018). The present review highlights seminal papers on skin‐resident memory T cells in the context of vitiligo and melanoma and addresses the potential significance of these cells for the treatment of vitiligo and melanoma.

## 2 FEATURES OF SKIN‐RESIDENT MEMORY T CELLS

### 2.1 Phenotypic characteristics of skin‐resident T_RM_ cells

**Figure 1:**
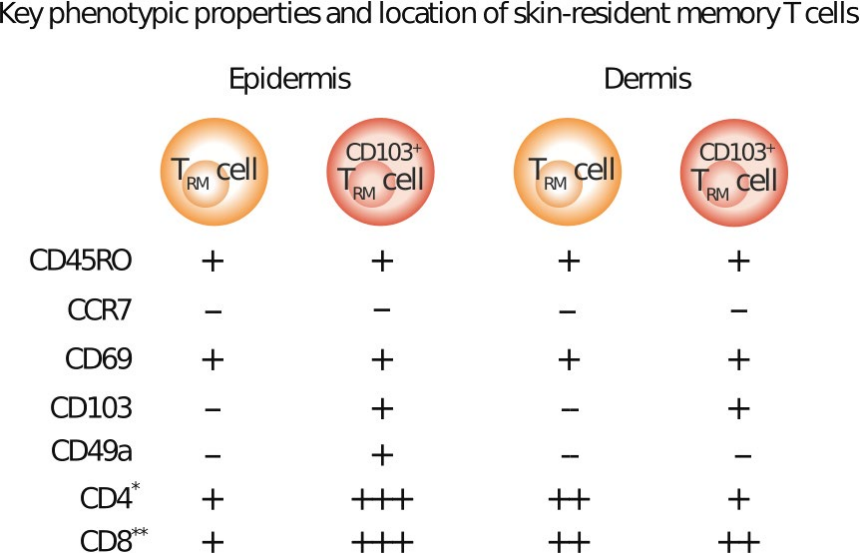
Key phenotypic properties and location of skin‐resident T cells. Phenotypic characteristics of skin‐resident memory T cells and distribution in human skin are shown. All memory T cells express CD45RO, but the absence of CCR7 and expression of CD69 and CD103 distinguish T_RM_ cells from circulating memory T cells. CD49a is found on epidermal CD69^+^ CD103^+^ CD8^+^ T cells only. CD69 and CD103 can be found on both CD4^+^ and CD8^+^ T cells, but at different levels. CD4^+^ T cells constitute approximately 75% of the lymphocytes present in both layers of the healthy human skin. However, there are twice as many CD103^+^ T_RM_ cells (both CD4^+^ and CD8^+^) in the epidermis, while in the dermis, the majority of T_RM_ cells are CD103^−^. −, no expression; + expression. Regarding CD4^+^ and CD8^+^: + represents low expression frequency; ++ medium expression frequency; +++ high expression frequency. * Fraction of CD69^+^CD4^+^ and CD69^+^CD103^+^CD4^+^ T_RM_ cells of the total CD4^+^ T_RM_ cell pool in either the epidermis or dermis are shown. **Fraction of CD69^+^CD8^+^ and CD69^+^CD103^+^CD8^+^ T_RM_ cells of the total CD8^+^ T_RM_ cell pool in either the epidermis or dermis are shown

The human skin contains approximately one million T cells per cm^2^, which amounts to almost 20 billion T cells in total (Clark et al., 2006). This is nearly twice as many T cells as those circulating in the blood. T_RM_ cells, like all memory T cells, can be distinguished from naïve T cells by expression of CD44, a marker of antigen experience. Furthermore, T_RM_ cells lack expression of CD62L and CCR7, which differentiates them from recirculating T_CM_ and T_MM_ cells (Figure 1). The chemokine receptor CCR7 interacts with CCL19 and CCL21, thereby helping T cells to migrate toward lymph nodes. As CCR7 expression is needed for T‐cell egress from peripheral tissues, CCR7^‐^ T cells in tissue can be considered tissue‐resident (Bromley, Thomas, & Luster, 2005; Bromley, Yan, Tomura, Kanagawa, & Luster, 2013; Debes et al., 2005). Another study showed that in normal skin under resting conditions, more than 90% of CCR7^‐^ CD62L^‐^ T cells co‐expressing the skin homing molecule CLA are skin‐resident (Clark et al., 2006).

To discriminate T_RM_ cells from T_EM_ cells, more phenotypic markers are needed. In human skin, 50%–70% of T cells express CD69 and CD103 (Watanabe et al., 2015). Although CD69 has been characterized as a T‐cell activation marker, it has been shown to be constitutively expressed by a subset of T cells within peripheral tissues under steady‐state conditions (Shiow et al., 2006) (Figure 1).

A subset of T_RM_ cells also expresses CD103, which is the α‐subunit of the α3β7 integrin receptor (Figure 1). In healthy human skin, its expression is most prominent on epidermal CD4^+^ and CD8^+^ T_RM_ cells, where it enables T_RM_ cell tethering within the epidermal compartment by binding to E‐cadherin, which is widely expressed by epithelial cells (Mackay et al., 2012). Nevertheless, binding to E‐cadherin is not required for skin residency (Nestle, Di Meglio, Qin, & Nickoloff, 2009). Although CD4^+^ and CD8^+^ CD103^+^ T_RM_ cells are less proliferative than CD103^‐^ T_RM_ cells, CD103^+^ T_RM_ cells have a larger effector cytokine production capacity (Watanabe et al., 2015). Relative proportions of resident and recirculating memory T (T_CIRC_) cells have been measured in highly immunocompromised NOD SCID IL‐2Rγ‐deficient (NSG) human‐engrafted mice and in lymphoma patients upon alemtuzumab treatment, which is an antibody specific to CD52 (expressed by T cells). Alemtuzumab depletes T cells by antibody‐dependent cellular cytotoxicity. This requires neutrophils and/or natural killer cells, which are relatively abundant in the circulation, but are rare in peripheral tissues. Alemtuzumab, therefore, only depletes T_CIRC_ cells, but not T_RM_ cells, which makes it possible to determine the relative proportions of both subsets. In healthy adult human skin, most T_RM_ cells are CD103^‐^ CD4^+^ and reside in the dermis. While CD103^+^ T_RM_, both CD4^+^ and CD8^+^, are more frequent in the epidermis, recirculating T cells are the minority among both CD4^+^ and CD8^+^ T‐cell populations in skin (Watanabe et al., 2015). The resident T‐cell populations in human skin thus differ in their migration compartments and functional capacities.

The α‐subunit of the α1β1 integrin receptor, CD49a (also known as very late antigen (VLA)‐1), was identified to delineate a subset of CD8^+^ T_RM_ cells in human skin epithelia that preferentially localize to the epidermis. These cells are poised toward IFN‐γ production and acquire high cytotoxic capacity upon IL‐15 stimulation (Cheuk et al., 2017). In the same study, CD49a^‐^ CD8^+^ T_RM_ cells excelled at IL‐17 production, and expression of CD49a was restricted solely to CD8^+^ T cells. Moreover, CD49a binds to collagen IV, a major component of the basement membrane between epidermis and dermis.

### 2.2 Tissue retention and transcriptional signatures shared by T_RM_ cells

Various molecular factors have been implicated in tissue retention. CD69 transcriptionally downregulates the sphingosine‐1‐phosphate receptor (S1P1), a G protein‐coupled receptor for sphingosine 1‐phosphate (S1P) (Mackay, Braun, et al., 2015). This limits egress of these memory cells out of tissues, showing that S1P1 downregulation is needed for long‐term residency of T_RM_ cells (Skon et al., 2013). Conversely, S1P1, through detection of its ligand S1P in the blood and lymph, is essential for naive lymphocytes to access the circulatory system from the thymus and lymph nodes (Matloubian et al., 2004). Effector T cells also use S1P1 to sense S1P gradients among tissues, lymph, and blood, thereby guiding entry in efferent lymphatics from lymphoid tissues (Spiegel & Milstien, 2011).

Expression of S1P1 can also be regulated by the transcription factor Kruppel‐like factor 2 (KLF2) (Skon et al., 2013). KLF2 was not expressed by CD69^+^ mouse memory CD8^+^ T cells isolated from non‐lymphoid tissues. Hence, T_RM_ did not express its target gene *S1pr1* (encoding S1P1), while forced S1P1 expression prevented establishment of T_RM_ cells. Furthermore, cytokines capable of inducing the CD69^+^ CD103^+^‐resident phenotype (including TGF‐β, IL‐33, and TNF) provoked KLF2 downregulation and thus downregulation of S1P1.

Expression of CD103 (or its ligand, E‐cadherin) by T_RM_ cells contributes to their maintenance in some non‐lymphoid tissues (Hofmann & Pircher, 2011), but is not a universal mechanism for residency retention in all tissues. For example, Casey et al. (2012) showed that while CD103 was required for maintenance of T_RM_ cells in the small intestinal intraepithelial lymphocyte population, it was found to be dispensable for memory cell establishment in the lamina propria lymphocyte population of the same organ.

Other factors involved in tissue retention include inflammatory cytokines such as transforming growth factor (TGF)‐β, interleukin (IL)‐33, and tumor necrosis factor (TNF)‐α. TGF‐β was shown to induce CD103 expression on mouse memory CD8^+^ T cells, and IL‐33 and TNF‐α were found to synergize with TGF‐β (Casey et al., 2012). This resulted in memory cells that adopted a resident phenotype (CD69^+^ CD103^+^) and indicates that tissues can intrinsically support differentiation of T_RM_ cells by the cytokine milieu. Stromal cells control tissue residency of memory T cells by expression of integrins, thereby regulating activation of TGF‐β (Mohammed et al., 2016). Moreover, TGF‐β and IL‐15 signaling were shown to be needed for development of T_RM_ cells in skin (Mackay et al., 2013). IL‐15 promoted formation and survival of T_RM_ cells in mice. IL‐15‐deficient mice had reduced T_RM_ cell formation, and this correlated with reduced Bcl‐2 expression, a prosurvival molecule, in CD103^+^ T_RM_ cells. Similarly, CD69 is rapidly induced in response to type 1 interferon (IFN) and suppresses S1P1 expression (Shiow et al., 2006).

It has been shown that T_RM_ has a transcriptional profile that is distinct from their memory T‐cell counterparts and includes transcription factors Hobit, Blimp1, and Runx3. In mice, the transcription factor Hobit is specifically upregulated in T_RM_ cells and, together with Blimp1, instructs tissue retention in different epithelial barrier tissues (Mackay et al., 2016). While Hobit was found to be essential for T_RM_ cell development, Blimp1 by itself was not, but synergized with Hobit. Also, Blimp1 was shown to initiate cytotoxic effector function, while Hobit was essential in the long‐term maintenance of granzyme B‐driven cytotoxicity (Kragten et al., 2018). The expression of Hobit is regulated by IL‐15 and the transcription factor T‐bet (Mackay, Wynne‐Jones, et al., 2015). In the absence of IL‐15, T_RM_ cells had decreased Hobit levels, and upon IL‐15 stimulation, activated CD8^+^ T cells upregulated Hobit expression in a T‐bet‐dependent manner (Mackay et al., 2016). Blimp1 expression, however, is not induced by IL‐15 or T‐bet. Its expression is regulated by the transcription factor Runx3 (D. Wang et al., 2018), which also promotes the expression of the T_RM_ retention markers CD69 and CD103 (Milner et al., 2017).

Data on human T_RM_ cell transcriptional profiles are now emerging. Compared to their circulating counterparts, CD8^+^ T_RM_ cells isolated from human lungs expressed high levels of *GZMB, IFNG, TNF,* and *NOTCH1* transcripts (Hombrink et al., 2016). Additionally, CD69^+^ memory cells from lung, spleen, and blood exhibited a transcriptional signature including CD103 and CD49a, chemokine receptors CXCR6 and CX3CR1, and immune checkpoint PD‐1 (Kumar et al., 2017). Despite similar core signatures with mouse T_RM_ cells, human T_RM_ cells lacked expression of Hobit.

## 3 IMMUNOSURVEILLANCE AND PROTECTION BY T_RM_ CELLS

Although T_RM_ cells do not recirculate throughout the body, they can migrate slowly within their environment. Antigen‐specific CD8^+^ T cells have been shown to crawl slowly between keratinocytes (Ariotti et al., 2012). This enables T_RM_ cells to identify antigen‐expressing target cells at different tissue locations within minutes to hours (Ariotti et al., 2014; Gebhardt et al., 2011). Their ability to scan the environment in which they persist after a primary infection is associated with enhanced pathogen detection upon reinfection by pathogens (Ariotti et al., 2012). T_RM_ cells are located in frontline sites of infection, such as the skin, lungs, and intestines and, therefore tend to respond rapidly to pathogen rechallenge.

Additionally, upon antigen resensitization T_RM_ cells trigger rapid innate and adaptive immune responses by secreting cytokines. Initially, T_RM_ cells can attract circulating memory T cells within hours by producing IFN‐γ (Schenkel, Fraser, Vezys, & Masopust, 2013). Moreover, T_RM_ cell‐derived IFN‐γ initiates an anti‐pathogen state at the local tissue site (Ariotti et al., 2014). At the same time, activated T_RM_ cells express TNF‐α, which is essential for dendritic cell maturation (Schenkel et al., 2014). Also, CD4^+^ and CD8^+^ CD69^+^ T cells are able to produce IL‐22, IL‐17, and anti‐inflammatory IL‐10. T_RM_ cells can thus trigger inflammation by pro‐inflammatory cytokines, but prevent excessive inflammation through IL‐10 (Kumar et al., 2017). Within 12 hr of local reactivation, T_RM_ cells express IL‐2, which leads to elevated levels of granzyme B secreted by both T_RM_ and natural killer cells (Schenkel et al., 2014). After local pathogen challenge, T_RM_ cells proliferate in situ and recruit memory T cells from the circulation, which subsequently undergo T_RM_ cell differentiation (Park, Zaid, et al., 2018). As a result, secondary T_RM_ cells are generated from preexisting T_RM_ cells, as well as from recirculating precursors. However, the preexisting T_RM_ cell populations are not displaced and remain in place in the tissue.

Despite their role in conferring protective immunity, T_RM_ cells can become pathologically activated and can cause tissue‐specific autoimmunity and inflammatory disease (Clark, 2015). The clinical characteristics of inflammatory lesions caused by T_RM_ cells manifest as fixed, delineated zones of lesions, with an abrupt cutoff from non‐lesional tissues. The pathogenic role of T_RM_ cells has been shown in many diseases, including psoriasis (Cheuk et al., 2014; Clark, 2011; Matos et al., 2017; Suarez‐Farinas et al., 2011) and cutaneous T‐cell lymphoma (Campbell et al., 2010). Commonly used treatments for psoriasis cannot fully deplete the pathogenic T_RM_ cells from skin lesions (Cheuk et al., 2014; Matos et al., 2017). This appears to explain why psoriatic lesions often reoccur at exactly the same anatomical location after therapy cessation.

## 4 THE ROLE OF T_RM_ CELLS IN AUTOIMMUNE VITILIGO

### 4.1 T_RM_ cells in the pathogenesis of vitiligo

Vitiligo is a common autoimmune disease, affecting approximately 1% of the general population. It results from the loss of epidermal melanocytes (Ezzedine, Eleftheriadou, Whitton, & van Geel, 2015). Genetic predisposition, environmental factors, and metabolic and immune alterations have been implicated in melanocyte destruction (Gauthier, Cario Andre, & Taieb, 2003; Picardo et al., 2015; Spritz, 2012). Previous studies have clarified the autoimmune etiology in human vitiligo. For example, vitiligo patients have melanocyte‐specific CD8^+^ T cells that are capable of killing melanocytes (van den Boorn et al., 2009; Ongenae, Van Geel, & Naeyaert, 2003; Palermo et al., 2001) and initiating antibody responses against melanocyte antigens, such as tyrosinase and TRP‐2 (Kemp, Gavalas, Gawkrodger, & Weetman, 2007).

**Figure 2:**
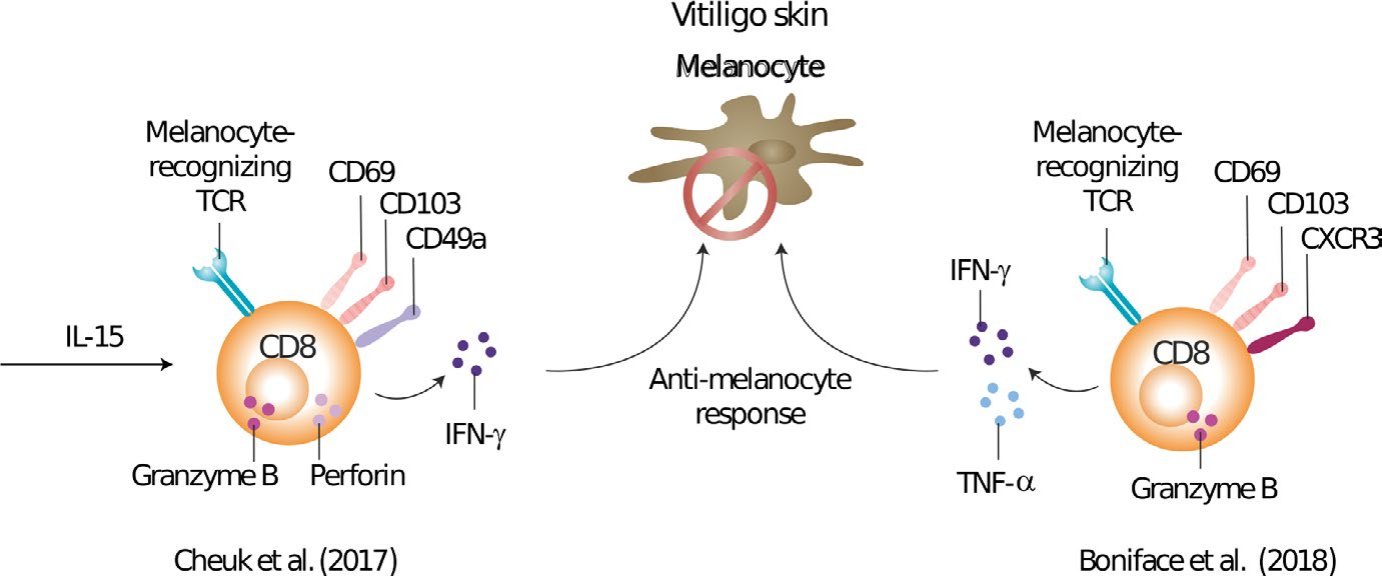
Resident memory T cells in vitiligo. The role of resident memory T cells in human vitiligo is shown. Firstly, Cheuk et al. (2017) reported an increase in CD49a^+^ T_RM_ cells in vitiligo skin, which produce IFN‐γ, granzyme B, and perforin upon IL‐15 stimulation. Furthermore, a substantial proportion of CD49a^+^ T_RM_ cells recognized melanocyte antigens, indicating a pathogenic role. Secondly, Boniface et al. (2018) showed vitiligo perilesional skin to be enriched with melanocyte‐specific CXCR3^+^CD8^+^ T_RM_ cells and CD8^+^ T_RM_ cells were poised for secretion of IFN‐γ and TNF‐α with moderate cytotoxic activity

Vitiligo lesions often recur at the same locations as those previously affected, suggesting that T_RM_ cells could be involved. A mouse model of melanoma‐associated vitiligo showed that both lesional and non‐lesional skin contained resident memory T cells, although they were preferentially localized in hair follicles containing white hairs (Malik et al., 2017). To induce melanoma‐associated vitiligo, mice were inoculated with B16 melanoma cells and depleted from regulatory T cells, after which the tumor was surgically removed. Vitiligo‐affected skin was shown to have CD8^+^ T cells recognizing tumor/self‐antigens and to exhibit a T_RM_ cell phenotype (CD44^hi^ CD62L^lo^ CD69^+^ CD103^+^). In line with this, autoreactive CD8^+^ T cells with a CD69^+^ CD103^±^ T_RM_ phenotype have been found in the skin of vitiligo patients (Boniface et al., 2018; Cheuk et al., 2017; Richmond, Strassner, Rashighi, et al., 2018; Richmond, Strassner, Zapata, et al., 2018) (Figure 2). Compared to healthy unaffected donor or psoriasis skin, lesional skin from vitiligo patients was shown to be enriched with CD49a^+^ CD103^+^ CD8^+^ (Cheuk et al., 2017) and CD69^+^ CD103^±^ CD8^+^ T_RM_ cells, independent of disease activity (Boniface et al., 2018). In the same study, it was suggested that the remaining CD8^+^ T_RM_ cells could possibly mediate disease flares or, alternatively, block repigmentation. CD8^+^ T_RM_ cells may prevent repigmentation by blocking either renewal of epidermal melanocytes or entry from the follicular reservoir of melanocyte precursors.

The chemokine receptor CXCR3 (the receptor for the chemokines CXCL9 and CXCL10) was shown to be important for epidermal localization of effector T cells and T_RM_ cell development (Mackay et al., 2013), which led to studies on CXCR3 expression in vitiligo patients. Perilesional skin from patients with progressive disease showed high CXCR3 expression (Bertolotti et al., 2014). Expression of CXCR3 was found on the majority of CD8^+^ T_RM_ cells in human vitiligo, including melanocyte‐specific cells, and these T_RM_ cells were poised for the secretion of IFN‐γ and TNF‐α (Boniface et al., 2018). These results indicated that targeting the CXCL9/10‐CXCR3 pathway could be an attractive strategy for the treatment of vitiligo. In line with this, another study reported an enrichment of IFN‐γ‐producing CD49a^+^ CD103^+^ CD8^+^ T_RM_ cells in vitiligo lesions, with a rapid granzyme B and perforin production response upon IL‐15 stimulation (Cheuk et al., 2017). Two studies showed that IFN‐γ and granzyme B are key cytokines in the pathogenesis of vitiligo because they induce melanocyte apoptosis (Harris et al., 2012; Yang et al., 2015). These results add to improved understanding of the autoimmune response in human vitiligo and suggest a profound role for CD8^+^ T_RM_ cells in human vitiligo, which explains the interest in targeting this cell subset in the treatment of vitiligo patients.

### 4.2 Therapeutic intervention in vitiligo

**Figure 3:**
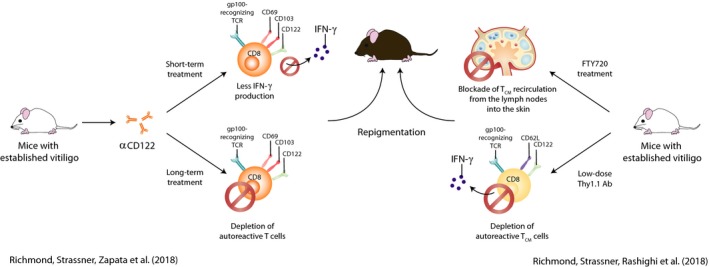
Therapeutic intervention in vitiligo. Potential therapeutic approaches for vitiligo are illustrated. Two murine studies showed that targeting T_RM_ cells can reverse disease in mice with established vitiligo. The left model shows T_RM_ cells express the IL‐15 receptor subunit CD122 and treatment with anti‐CD122 antibody led to repigmentation. Short‐term treatment led to less IFN‐γ production by T_RM_ cells, and long‐term treatment depleted autoreactive T_RM_ cells and other memory T‐cell pools. The right model shows that T_RM_ and T_CM_ cells cooperate to maintain vitiligo. Treatment of mice with FTY720, which limits T‐cell access to the skin, or low‐dose Thy1.1 antibody, which depletes T_CM_, resulted in repigmentation

Based on the fundamental pathogenic role of T_RM_ cells in human vitiligo, novel strategies specifically targeting T_RM_ cells may improve the treatment outcome of vitiligo. T_RM_ cell formation has been shown to be highly dependent on IL‐15, and IL‐15 promoted T_RM_ cell function ex vivo (Adachi et al., 2015; Mackay et al., 2013). A subsequent study therefore looked at IL‐15 signaling as a therapeutic target for vitiligo (Richmond, Strassner, Zapata, et al., 2018). Treatment with anti‐CD122 antibody, a subunit of the IL‐15 receptor on human and mouse T_RM_ cells, was shown to reverse disease in mice with established vitiligo (Figure 3). A 2‐week short‐term treatment decreased IFN‐γ production, while an 8‐week long‐term treatment depleted autoreactive CD8^+^ T_RM_ cells. These findings indicate that targeting IL‐15 signaling via CD122 may be an effective strategy to treat vitiligo and possibly other T_RM_ cell‐mediated diseases. T_RM_ cell survival and function also depend on the uptake of exogenous lipids and on their oxidative metabolism (Pan et al., 2017). Future studies targeting this pathway might reveal if it can affect or even deplete T_RM_ cells from peripheral tissues.

Besides melanocyte‐specific T_RM_ cells, autoreactive T_CM_ cells have been found in the blood of vitiligo patients (van den Boorn et al., 2009; Ogg, Rod Dunbar, Romero, Chen, & Cerundolo, 1998), and these cells have the potential to home to the skin. However, the functional capacity of T_CM_ has remained unknown. A study examining the functional relationship between T_CM_ and T_RM_ in a vitiligo mouse model reported that T_CM_ cells cooperate with T_RM_ cells to maintain disease (Richmond, Strassner, Rashighi, et al., 2018) (Figure 3). Both subsets recognized self‐antigen and secreted IFN‐γ and chemokines. Gp100‐specific CD69^+^ CD103^+^ CD8^+^ T_RM_ cells produced CXCL9 and CXCL10, which are chemokines recognized by CXCR3, potentially to recruit T_CM_ cells toward melanocytes in the skin. Treatment of mice with FTY720, as a means of blocking T‐cell access to the skin, or low‐dose Thy1.1 antibody, to deplete T_CM_, resulted in reversal of disease. This study suggests that circulating and resident T cells cooperate in vitiligo pathogenesis. However, the extent of such a relationship between circulating and resident memory T‐cell subsets in human vitiligo remains unclear. Better understanding of this relationship may give clues on how pathogenic T cells can most effectively be targeted in vitiligo.

## 5 SKIN‐RESIDENT T‐CELL RESPONSES IN MELANOMA

### 5.1 Prognostic significance of resident memory‐like tumor‐infiltrating lymphocytes (TILs)

**Figure 4:**
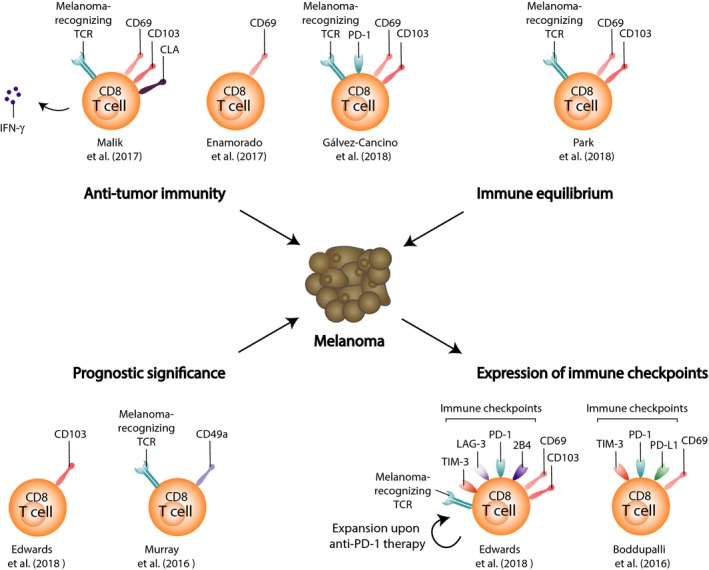
Resident memory T cells in melanoma. Skin‐resident memory T cells can have various roles in melanoma; (1) T_RM_ cells can mediate anti‐tumor immunity, upon vitiligo induction strategies or intradermal vaccine administration (murine data), (2) CD8^+^ T_RM_ cells promote a melanoma–immune equilibrium in the epidermis (murine transplanted melanoma model), (3) tumor infiltration of T cells expressing either CD103 or CD49a, frequently co‐expressed with CD69 and CD103, is correlated with improved survival of melanoma patients, and (4) metastatic melanoma patients show expression of the immune checkpoints PD‐1, LAG‐3, 2B4, and TIM‐3 on intratumoral T_RM_ cells, with or without CD103 co‐expression

While T_RM_ cells have been widely characterized in viral infections, their role in mediating tumor immunity is not yet fully known. Studies analyzing the infiltration of T_RM_ cells in human tumors have shed some light on their relevance in anti‐tumor immunity. Tumor infiltration of CD8^+^ T cells exhibiting a resident phenotype (CD69^+^ CD103^+^ and/or CD103^+^) correlates with a more favorable prognosis for various human cancers (Djenidi et al., 2015; Koh et al., 2017; Wang et al., 2015; Webb, Milne, Watson, Deleeuw, & Nelson, 2014). Similar correlations were shown for human melanoma (Edwards et al., 2018; Murray et al., 2016) (Figure 4). CD103^+^ CD8^+^ T cells, residing in the tumor microenvironment, were strongly correlated with increased melanoma‐specific survival in immunotherapy‐naïve stage III melanoma patients (Edwards et al., 2018). High CD103^+^ CD8^+^ T_RM_ cell counts led to a 5‐year survival rate of 50% compared to 20% in those with lower counts. Also, expression of CD49a by vaccine‐induced CD8^+^ T cells was shown to predict a prolonged overall and disease‐free survival in stage III/IV melanoma patients (Murray et al., 2016) (Figure 4). CD49a^+^ CD8^+^ TILs were found to be enriched in human melanoma metastases in various peripheral tissues. Most interestingly, CD49a was frequently co‐expressed with CD69 and CD103, and in vivo blockade of CD49a or CD103 in a C57BL/6 melanoma mouse model significantly impaired control of subcutaneous B16‐OVA tumors, supporting the notion of T_RM_ cell‐mediated anti‐tumor immunity. CD49a^+^ B16‐OVA‐derived T_RM_ cells produced higher levels of IFN‐γ and granzyme B and exhibited a high activation status, which was even more prominent in the CD103^+^ subset. Moreover, in human melanoma, local IL‐15 levels strongly correlated with tumor‐resident CD8^+^ T‐cell numbers, and high IL‐15 levels were associated with a more favorable prognosis (Edwards et al., 2018). IL‐15 seems to be essential in retaining T cells within the tumor microenvironment, indicating that IL‐15 is worthy of further investigation, as supported by the murine vitiligo data discussed previously (Richmond, Strassner, Zapata, et al., 2018).

### 5.2 Expression of immune checkpoints by resident memory‐expressing TILs

Upregulation of immune checkpoints on TILs has emerged as a major barrier to effective anti‐tumor immunity. Interestingly, not all TILs express immune checkpoints. Identifying which subpopulations among tumor‐infiltrating immune cells—defined both phenotypically and functionally—express immune checkpoints is important in evaluating anti‐tumor immunity. Counterintuitively, in human melanoma metastases, tumor‐associated CD8^+^ T cells with a T_RM_ phenotype were shown to express the highest levels of immune checkpoints, such as PD‐1 and TIM‐3, independent of CD103 expression (Boddupalli et al., 2016) (Figure 4). In the same study, TILs simultaneously produced less cytokines, which is consistent with an exhausted phenotype (Baitsch et al., 2011). Another study reported that mainly the CD103^+^ CD8^+^ subset within TILs expressed high levels of PD‐1, LAG‐3, 2B4, and TIM‐3 (Edwards et al., 2018) (Figure 4). Upon anti‐PD‐1 therapy, CD103‐expressing CD8^+^ T_RM_ cells significantly expanded, suggesting that these cells have been released from the negative effect of PD‐1 checkpoint signaling by anti‐PD‐1 therapy. Tumor‐resident TILs may thus represent a major target for immune checkpoint blockade. However, these findings were based on markers that are redundant with other T‐cell states. For example, CD69 and PD‐1 can be co‐expressed as a result of recent antigen stimulation. Similarly, TGF‐β has been shown to induce expression of CD103 on CD8^+^ T cells (El‐Asady et al., 2005) and TGF‐β is often produced within the tumor environment (Thomas & Massague, 2005). It therefore remains unclear whether inhibitory checkpoint molecules are particularly enriched on resident memory‐expressing TIL or whether resident cell markers are expressed as result of environmental factors or antigen stimulation.

Another consideration is that less immune checkpoint expression is found on T_RM_ cells in normal skin or during autoimmune response or infection. In the B16 mouse model of melanoma‐associated vitiligo, it was shown that cutaneous tumor/self‐antigen‐specific CD8^+^ T_RM_ cells located within depigmented hair follicles lacked PD‐1 and LAG‐3 expression (Malik et al., 2017). Likewise, skin CD8^+^ T_RM_ cells lacked PD‐1 expression in a murine model of viral infection (Jiang et al., 2012). In a vitiligo mouse model, however, autoreactive CD8^+^ T_RM_ cells did express PD‐1 (Richmond, Strassner, Rashighi, et al., 2018). This indicates that expression of immune checkpoints has not been fully elucidated and requires more attention in future research.

### 5.3 Clinical implications of resident memory‐like TILs

Current data suggest an important role for TILs that express T_RM_‐associated markers in providing anti‐tumor immunity and their potential as biomarkers. Immune checkpoints seem to be enriched particularly on T_RM_ cells, suggesting that the T_RM_ subset of TILs may be the major target for immune checkpoint blockade. Hence, cancer immunotherapy vaccination strategies should aim at priming tumor‐reactive T_RM_ cell subsets, which could synergize with immune checkpoint blockade.

Human metastatic lesions are enriched with CD8^+^ T_RM_‐like cells (Boddupalli et al., 2016), and adoptive transfer of resident memory‐like TILs might be a promising therapeutic option to melanoma patients. However, individual metastasis in the same patient may contain a distinct repertoire of T_RM_‐like cells. Sequencing of the T‐cell receptor (TCR) revealed interlesional heterogeneity of TILs, which was also found in the resident T‐cell population (Boddupalli et al., 2016). This heterogeneity was not due to variance in mutations or neoepitopes in tumor cells. Consequently, patients may experience mixed responses, with some tumor lesions regressing and others progressing, as occasionally observed following immunotherapy. It is therefore logical to explore adoptive transfer of T_RM_‐like TIL isolated from multiple lesions, as this may provide a more diverse repertoire. However, considering the limited sample number in the study of Boddupalli et al. (2016), interlesional heterogeneity should be confirmed in larger studies.

### 5.4 Anti‐tumor immunity by T_RM_ cells

Despite the great interest in immunotherapy, its clinical success still requires substantial optimization. To improve the efficacy of cancer immunotherapy, it is important to induce a potent effector response together with a stable, functional memory response, thus protecting the patients from cancer recurrence or relapse. Mouse models of melanoma have shown that tumor‐specific T_RM_ cells can protect against highly aggressive melanoma. CD8^+^ T_RM_ cells driven by a model of autoimmune vitiligo were shown to inhibit melanoma growth in a CD103‐dependent manner (Malik et al., 2017). Also, infecting the skin with recombinant vaccinia virus expressing full‐length ovalbumin (OVA) protein generated CD8^+^ T_CIRC_ and T_RM_ cells that delayed the growth of OVA‐expressing melanoma (Enamorado et al., 2017) (Figure 4). Intraperitoneal vaccination, which generates T_CIRC_ only, or FTY720 treatment, which blocks T‐cell access to the skin, revealed that either T_CIRC_ or T_RM_ cells were sufficient for protection against B16‐OVA re‐challenge in the skin, but that the presence of T_RM_ cells improved anti‐tumor efficacy (Enamorado et al., 2017). Gálvez‐Cancino et al. (2018) showed that intradermal administration of vaccines, which are known to induce strong CD8^+^ T‐cell responses, efficiently induced T_RM_ cell responses against tumor antigens and self‐antigens (Gálvez‐Cancino et al., 2018) (Figure 4). Moreover, growth of cutaneous melanoma tumors was strongly suppressed, independently of circulating CD8^+^ T cells and other adaptive immune cells. Similarly, CD8^+^ T_RM_ cells promoted a melanoma–immune equilibrium in the epidermal layer of the skin (Park, Buzzai, et al., 2018) (Figure 4). In the B16/Bl6 mouse melanoma model, approximately 40% of mice that received epicutaneous inoculation of B16 melanoma cells remained free of macroscopic tumor growth. Tumor cells were dynamically surveyed by CD69^+^ CD103^+^ CD8^+^ T_RM_ cells, and T_RM_ cell responses were observed more often and at higher densities in peritumoral skin than in the skin of tumor‐bearing mice. In line with the findings of Enamorado et al. (2017), melanoma development was also suppressed in the majority of mice, irrespective of depletion of T_CIRC_ cells, but protection was most pronounced in mice harboring both T_CIRC_ and T_RM_ cells. These studies clearly affirm the potential of intradermal vaccine‐induced T_RM_ cells to achieve potent protection against skin cancer. To effectively protect against malignancies, cancer vaccines should therefore evoke potent T_RM_ cell responses within the tissue.

## 6 CONCLUSIONS

Emerging evidence has shown that non‐recirculating T_RM_ cells constitute a large fraction of the memory T‐cell pool and are involved in controlling various infectious diseases, such as cancer, or in mediating autoimmunity. The growing appreciation that T_RM_ cells are central players in immunity to vitiligo and melanoma has led to increased interest in T_RM_ cells as promising targets for future vaccines and immunotherapies.

T_RM_ cells are likely to have a prominent role in disease development and flare‐up in human vitiligo. Therefore, targeting T_RM_ cells appears to be an attractive treatment strategy. Blocking the generation, maintenance, and coordination of T_RM_ cells efficiently inhibits melanocyte killing in mice models. Future trials in patients will provide important insights into targeting T_RM_ cells for the treatment of human vitiligo.

Although not fully confirmed by all studies so far, inhibitory immune checkpoints appear to be particularly enriched on cells with T_RM_ cell properties in human melanoma. At the same time, however, their expression on T_RM_ cells in the context of autoimmune vitiligo remains unstudied. In vitiligo, the autoimmune reaction is not downregulated, but remains present; future studies might therefore clarify whether immune checkpoint expression is possibly dispensable on vitiligo‐associated T_RM_ cells.

The evidence on the contribution of T_RM_ cells in cancer suppression also shows how the manipulation of T_RM_ cells can be beneficial in optimizing the anti‐tumor immunity. Vaccination strategies have successfully generated T_RM_ cell populations that have effectively suppressed tumor growth in mouse models of melanoma. However, developing these therapies will require additional experimental studies to obtain more insight into the exact phenotype and function of T_RM_ cells in mice and humans. Furthermore, to validate data from mouse experiments in human clinical trials, it is crucial to study the potential of targeting T_RM_ cells in human disease.

The research highlighted in this review has focused on CD8^+^ T_RM_ cells as key mediators of anti‐tumor immunity. However, the role of CD4^+^ T_RM_ cells in immunity to cancer remains undefined. Future studies should clarify whether tumor immunity benefits from local helper T_RM_ cells, and whether regulatory T_RM_ cells are detrimental to this immunity. Moreover, studies on vitiligo have not reported data on CD4^+^ helper or regulatory T_RM_ cell subsets either. With more knowledge becoming available on the involvement of T_RM_ cells in autoimmunity and cancer, future research will hopefully overcome barriers to effectively block or to promote effective responses of T_RM_ cells to vitiligo and melanoma.
